# SeqX: a tool to detect, analyze and visualize residue co-locations in protein and nucleic acid structures

**DOI:** 10.1186/1471-2105-6-170

**Published:** 2005-07-12

**Authors:** Jan C Biro, Gergely Fördös

**Affiliations:** 1 Homulus Foundation, 88 Howard, #1205, San Francisco, CA 94 105-1649, USA; 2 Department of Control Engineering and Information Technology, Budapest University of Technology and Economics, Magyar tudósok körútja 2. Budapest 1117, Hungary

## Abstract

**Background:**

The interacting residues of protein and nucleic acid sequences are close to each other – they are co-located. Structure databases (like Protein Data Bank, PDB and Nucleic Acid Data Bank, NDB) contain all information about these co-locations; however it is not an easy task to penetrate this complex information. We developed a JAVA tool, called SeqX for this purpose.

**Results:**

SeqX tool is useful to detect, analyze and visualize residue co-locations in protein and nucleic acid structures. The user

a. selects a structure from PDB;

b. chooses an atom that is commonly present in every residues of the nucleic acid and/or protein structure(s)

c. defines a distance from these atoms (3–15 Å). The SeqX tool detects every residue that is located within the defined distances from the defined "backbone" atom(s); provides a DotPlot-like visualization (Residues Contact Map), and calculates the frequency of every possible residue pairs (Residue Contact Table) in the observed structure. It is possible to exclude +/- 1 to 10 neighbor residues in the same polymeric chain from detection, which greatly improves the specificity of detections (up to 60% when tested on dsDNA). Results obtained on protein structures showed highly significant correlations with results obtained from literature (p < 0.0001, n = 210, four different subsets). The co-location frequency of physico-chemically compatible amino acids is significantly higher than is calculated and expected in random protein sequences (p < 0.0001, n = 80).

**Conclusion:**

The tool is simple and easy to use and provides a quick and reliable visualization and analyses of residue co-locations in protein and nucleic acid structures.

**Availability and requirements:**

SeqX, Java J2SE Runtime Environment 5.0 (available from [see Additional file 1] ) and at least a 1 GHz processor and with a minimum 256 Mb RAM. Source codes are available from the authors.

## Background

Specific protein-protein and protein-nucleic acid interaction are in the focus of many biochemical studies. The exact nature of these interactions is not known. Some scientists argue that the macromolecular interactions are determined by long sequence domains that are involving many residues (amino acids and nucleotides), while others found that there is some degree of specificity already on a single residue level, i. e. some residue pairs are preferentially co-located on interacting interfaces. The existence of preferred residue pairs within, as well as between, macro-molecular structures are supported by numerous statistical analyses of protein-RNA [1] regulatory protein-DNA [2], restrictions enzyme-DNA cut site [3], protein-protein [4-8] structures and interfaces. Although many studies are performed for statistical analyses of residue co-location, it was not possible for us to find a publicly available tool for this purpose. We found only a reference for the existence of a commercially available tool, the QUANTA modeling software [9,10].

## Implementation

**Figure 1:**
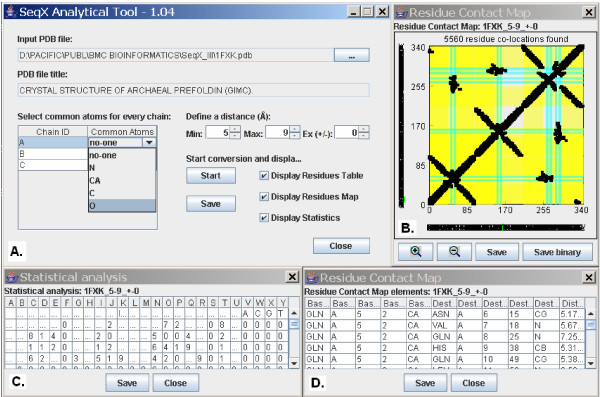
**Main features of the SeqX analytical tool**. A: Main Window, B: a dot-plot like Residue Contact Map, C: Statistical Analyses of residue co-location in a matrix format and D: the details of the Residue Contact Map elements.

Any structure files (.pdb) may be selected for analyses from the main window. (Figure 1). The tool automatically provide the title of the selected PDB file, a list of sequences present in the file and a list of every common atom in the residues of the respective sequences. These possible backbone atoms are N, CA: C_alpha_, C and O in proteins; and P, O1P: O_1_P, O2P: O_2_P, O5*: O_5_', C5*: C_5_', C4*: C_4_', O4*: O_4_'. C3*: C_3_', O3*: O_3_', C2*: C_2_', C1*: C_1_', C5: C_5_, C6: C_6_, N1: N_1_, C2: C_2_,, N3: N_3 _and C4: C_4 _in nucleic acids. It is possible to exclude one or more sequence from analyses by selecting the "no-one" option in the Common Atoms list. The user is asked to define a spherical space around the selected core atoms by choosing a minimum and maximum detection radius around these atoms (between 0 to 15 Ångströms). It is usually not interesting to detect residue co-locations related to neighbor residues in the same sequences. Therefore it is possible to exclude up- and downstream neighbors in the same sequence. (Ex +/-: 0–10). The program ignores terminal residues if they are annotated as HETATM i.e. non-standard residues. The SeqX program makes a list of atoms (and the corresponding residues) which are located within the defined radius around the pre-selected common atoms and are not excluded as neighbor residues. This list is accessible as a Residue Table that contains the Residue Contact Map elements. The atomic distances are calculated by the Pythagoras theses. The results of these analyses are visualized in a Residue Contact Map and summarized in a statistical table. The Residue Contact Map is a dot-plot like graph where every residue in every sequence in the PDB structure is compared to each other, and residue co-locations are indicated by a square. The color of the squares indicate the type of molecular contacts (blue: nucleic acid – nucleic acid, red: protein – nucleic acid, black: protein – protein).

The main features of the protein secondary structure are indicated by background colors (blue/green: beta sheet, yellow: alpha helix, gray: turn), if they are annotated (not always) in the pdb source files.

It is possible to zoom in the center of the Map and move it into optional directions (using the mouse). Primary structure (the sequence) is available along the coordinates. If the sequence is too long, it is necessary to zoom in the Map to make the sequence readable.. Protein sequence is indicated with the 20 one-letter codes (capital letters), while the nucleic acid sequence with the a, t/u, g, c letters. Clicking on any co-locations highlights the corresponding 2 letters in the sequences (green letter coloring).

A simple statistical analysis is performed and the number of every possible residue combinations is listed in a Residue Contact Table. It is possible to save the results of the analysis in JPG and XLS (or similar) files. It is also possible to save even the Residue Contact Map in binary form and XLS format for future statistical processing ("Save binary" saves the map as 0 a 1 numbers).

## Results

**Figure 2:**
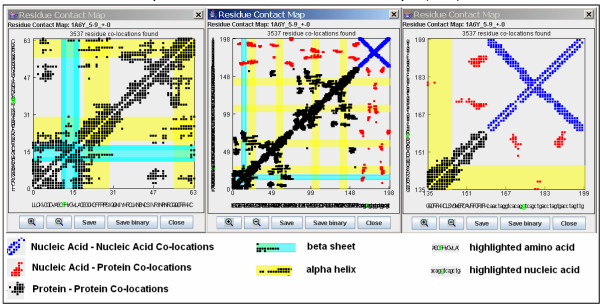
**SeqX: Residue co-locations in a nucleo-protein complex (1A6Y)**. Residue Contact Map of a nucleo-protein complex (1A6Y) was generated by SeqX. Residue co-locations were detected within 6–9 Å radiuses from C_alpha _amino acid and C_1_' nucleotide atoms and it was not necessary to use the closest neighbor exclusion option, Ex (+/-): 0. The different kind of molecular co-locations and the main features of the protein secondary structure is color coded. It is possible to zoom in the central portion of the map (middle) and navigate it in optional directions using the mouse (left and right part of the figure). The primary structure (sequence) is readable along the x and y axis in the magnified maps and it is possible to highlight individual co-locations (green letters).

**Figure 3:**
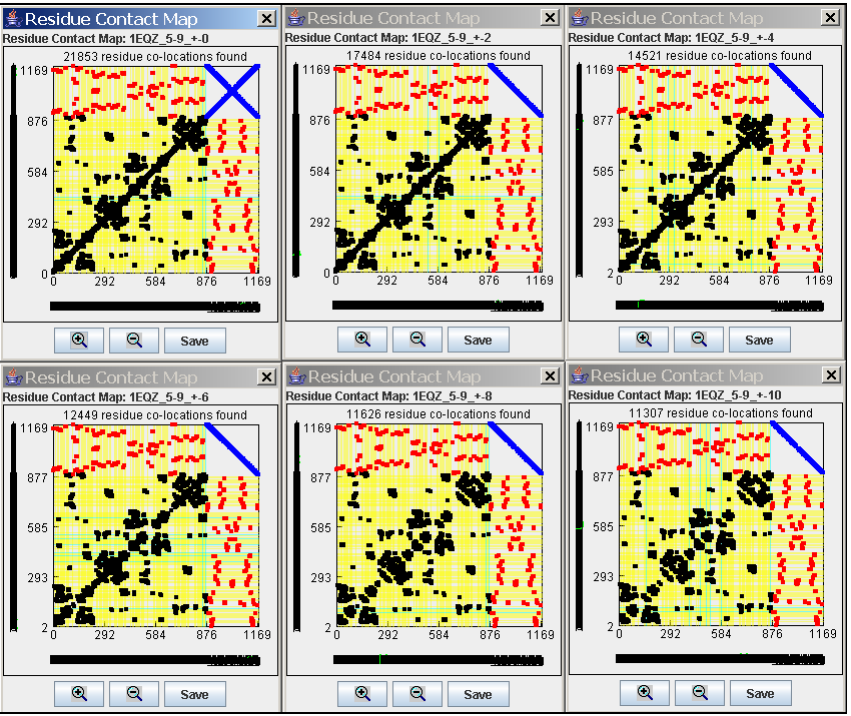
**Effect of neighbor residue exclusion on the residue contact map of a nucleoprotein complex**. Residue Contact Maps of 1EQZ nucleoprotein structure using constant 5–9 Å radius around C_alpha _and C_1_' atoms but varying the Ex +/- value from 0 to 10 Å Note the successive disappearance of the right diagonal line which corresponds to co-locations of neighbor residues in the same strand.

The Residue Contact Map provides a 2D dot-plot like graph of residue co-locations in protein, nucleic acid or nucleoprotein complexes. (Figure 2). This plot is simple and as easy to understand as any other dot-plot. The main right diagonal line corresponds to residue co-locations in the same polymeric chain (neighbors) and it is possible to eliminate by neighbor exclusion. (Figure 3).

The Residue Contact Table contains all possible residue combinations (20 × 20 amino acid to amino acid, 4 × 4 nucleic acid to nucleic acid and 20 × 4 amino acid to nucleic acid combinations) and lists the frequency of theses co-locations in the observed structure. Some of the listed co-locations are specific (true) while other is aspecific (false) co-locations.

It is possible to estimate the specificity of the results only in the case of nucleic acids where the Watson-Crick base pairs are known to be specific co-locations. The Residue Contact Table provides data for the 16 (4 × 4) different type of nucleic acid base co-locations, however it is known that only adenine-thymine (a-t, t-a) and guanine-cytosine (g-c, c-g) co-locations indicate true (T) and specific base-pairs, while the 8 other pairs are false (F).

**Figure 4:**
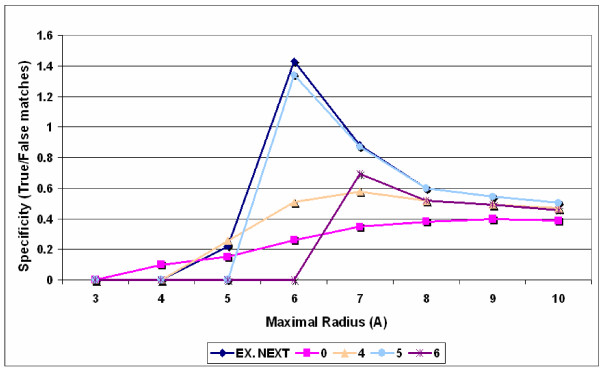
**Effect of detection radius on specificity of SeqX (B-DNA)**. The number of nucleotide co-locations was monitored by SeqX using a Palindromic 146 Base-pairs long B-DNA Fragment (in 1AOI). The minimal detection radius varied from 0–6 Å (from the C_1_' atom), (inserted legend) while the maximal radius was 1–10 Å (X-axis). The ratio of the detected true and false Watson & Crick base-pairs was regarded to be the specificity of the measurements. EX.NEXT indicates when the closest base neighbors on the same strand were automatically excluded from the detection by the tool.

**Figure 5:**
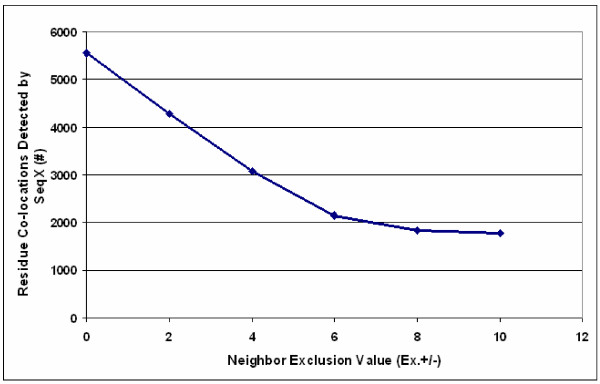
**Residue co-location vs. neighbor exclusion value**. Residue co-locations were detected by SeqX in a helical protein (1FXK). The detection radius was kept constant (5–9 Å) around the C_α _atoms but the neighbor exclusion value (EX +/-) was varying.

**Figure 6:**
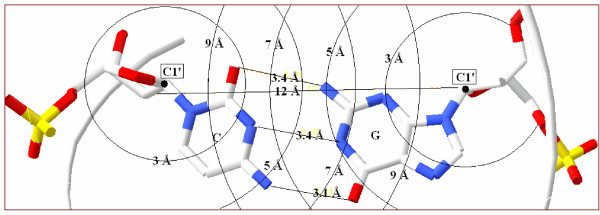
**Atomic distances in dsDNA (upper view)**. The distance between C_1_' atoms and a cytosine (C) – guanine (G) base pair is indicated. Circles are drawn around C1' atoms (radius: 3-5-7-9 Å).

**Figure 7:**
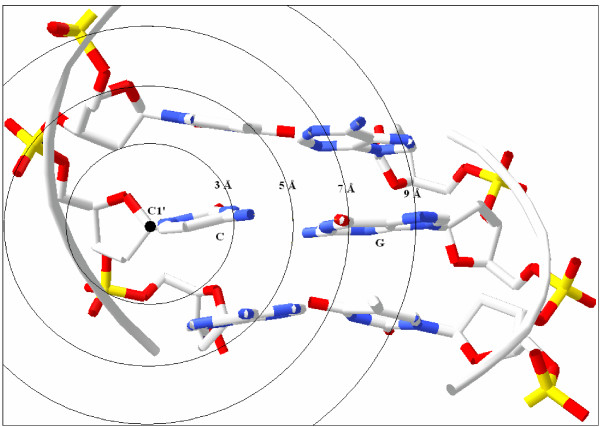
**Atomic distances in dsDNA (side view)**. Three base pairs are indicated in a dsDNA. Circles are drawn around C_1_' atom of a cytosine (C) using 3-5-7-9 Å radius. G: Guanine.

**Figure 8:**
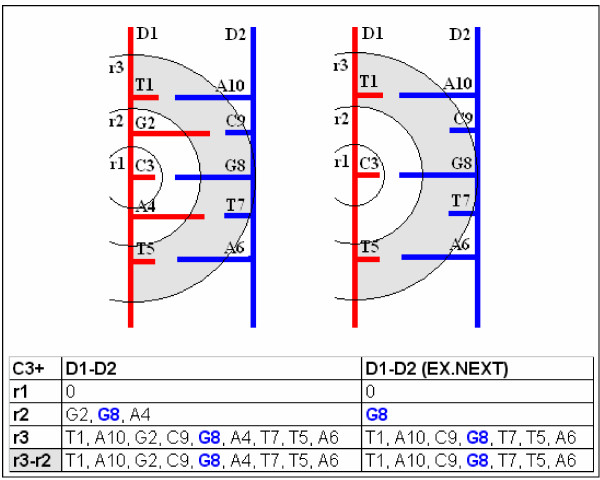
**Effect of monitoring radius on detection of residue co-locations in a dsDNA**. Residue Co-locations are indicated in a dsDNA (D1-D2). Circles show distances (r1, r2, r3) from the C_1_' atom of a cytosine (C3). Residue co-locations ware detected by SeqX tool and summarized in the table. The number of detected thymine (T), guanine (G), adenine (A) depended on the radius. Shaded circle indicate area between r3 and r2 radius. G8, the expected complementary base to C3 was most selectively found using r2 radius and Ex. +/- 1 option which excluded closest neighbors to C3 in D1 from the detection.

The estimated specificity of the SeqX tool on dsDNA is up to 60% (T/F ~ 1.4), (Figure 4). The specificity is greatly improved by proper distance selection and exclusion of residue neighbors. (Figure 5). It is easy to explain the reason for these observations. (Figure 6, 7, 8).

**Figure 9:**
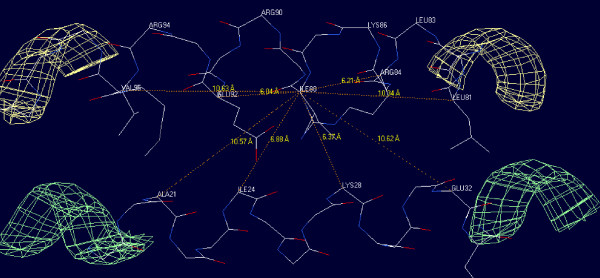
**Atomic distances in a protein structure of two parallel alpha helices**. Distances between C_-α _atoms are indicated around ILE88. The distance to the -4^th ^ARG84is 6.2 A and to the +4^th ^GLU92 is 6.0 A. This indicates that Ex. +/- 4 might be necessary to exclude the aspecific neighbor effects.

It is more difficult to find optimal SeqX parameters for studying residue co-locations in- and between proteins. In contrast to the DNA it is not known which (if any) amino acid pairs represent specific residue co-locations. Furthermore some protein structures are very compact and, for example, in the case of alpha helical proteins many amino acid neighbors might interfere with the specificity of the detection (Figure 9) and the exclusion of more than one neighbor is necessary to improve the specificity of the detection.

We found that detection radius between 5–9 Å and exclusion of +/-8 neighbors gives the best results for analyzing alpha helical protein structures.

**Figure 10:**
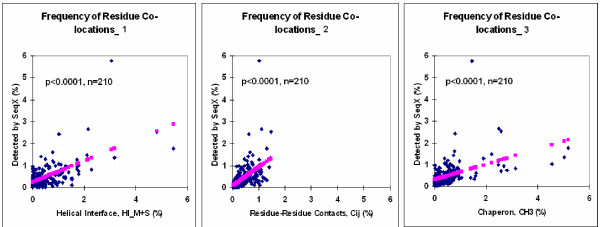
**Frequency of residue co-locations_1-3**. A subset of amino acid co-locations (34630) were identified by SeqX in proteins which contained preferentially alpha helices. The frequency of these co-locations were compared to the frequency of residue co-locations with data from literature such as (1) Helical Interface, HI_M+S [8]; (2) Residue-Residue Contacts, Cij [6] and (3) chaperon, CH3 data [5].

A real specificity estimation is not possible to do on protein sequences (not even in receptor-ligand structures), because the amino acids are not known to be complementary to each other. Therefore the frequency of amino acid co-locations found by SeqX (preferentially in alpha helical proteins) is compared to the frequency of residue co-locations data from literature [5,6,8]. Our results showed highly significant correlation to data from the literature (p < 0.0001, n = 210). (Figure 10).

Some cautious and preliminary estimation is still possible even for the specificity of detected residue co-locations in protein structures. Namely, it is known from physico-chemical studies, that some amino acids are attractive while others are repulsive to each other. The known physico-chemical laws suggest that pair-formation (co-location) is probably preferred between amino acids having similar hydrophobicity or different charge, while pair-building between amino acids with different hydrophobicity or similar charge are strongly prohibited.

To test this assumption we generated a pool of artificial random protein sequences by translating randomized nucleic acid sequences. The nucleic acids contained equal amount of each nucleotide bases (4 × 25%) and, by that way, the average frequency of amino acids in the translated artificial proteins became very similar to the amino acid frequency of the entire human proteome.

The residue co-locations within and between these sequences are determined by statistical lows if we assume that the spatial mobility of the residues in these proteins is free and independent of each other. The calculated probability of any residue co-locations (P_ab_) will be P_ab _= n_a_n_b_/T^2^, T = n_a_+n_b_...+n_20 _wher n is the number of a given amino acid. The calculated relative frequency of a given co-locating pair (C_ab_) is proportional to P_ab _and might be calculated by the C_ab _= P_ab_/ (P_ab_+...P_xy_) 100 formula, where x and y indicate any of the 20 possible amino acids and the number of xy pairs is 400.

**Figure 11:**
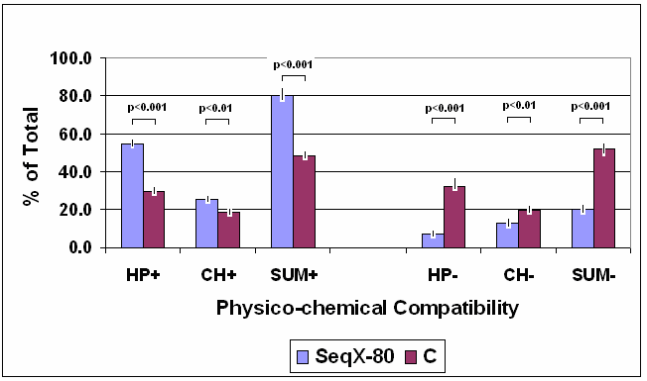
**Real vs. calculated residue co-locations**. The relative frequency of real residue co-locations were determined by SeqX in 80 different protein structures and compared to the relative frequency of calculated co-locations in artificial, random protein sequences (C). The 200 possible residue pairs provided by the 20 amino acids were grouped into 4 subgroups regarding their physico-chemical compatibility to each other i.e. favored (+) and un-favored (-) regarding hydrophobicity and charge. (HP+: hydrophobe – hydrophobe and lipophobe – lipophobe; HP-: hydrophobe – lipophobe; CH+: positive – negative and hydrophobe – charged; CH-: positive-positive and negative -negative and lipophobe – charged interactions). The bars represent the mean +/- S.E.M. (n = 80 for real structures and n = 10 for artificial sequences). Student's t-test was applied to evaluate the results.

The relative frequency of physico-chemically favored co-locations is significantly higher (and the relative frequency of un-favored co-locations is significantly lower) in real protein structures (determined by SeqX) than it is calculated for random interactions (p < 0.001, n = 80 and n = 10, respectively). (Figure 11.)

This example indicates that the number of false positive (un-favored) co-locations is about 20% and the specificity of SeqX methods for proteins might be as much as ~80% However this is a very crude estimate, because the number of true co-locations is not surely known.

## Discussion

To understand the nature of specificity of macromolecular interactions is a major challenge in bioinformatics. We were successful in providing evidence to support the view that some degree of specificity already exists on residue level [3]. Therefore we decided to continue our studies of frequency analyses of residue co-locations in nucleoprotein structures. The SeqX tool is specifically designed for this purpose. The 2D Residue Contact Map is a simple and easy to understand display of nucleic acid and protein structures. There are some very sophisticated analytical tools which also even incorporate this feature, like MOLTALK [11], STING Millennium [12], STRIDE [13] MolSurfer [14] MOLPROBITY [15]. The major advantage of this approach is its simplicity. The effective usage of 3D tools and learning the "3D thinking" usually requires lengthy training which often is not affordable for general bioinformaticians. We have further developed the concept of Residue Contact Map and added many new features that are not present in existing tools. Such features are

1., The option to choose different backbone atoms (in addition to the conventional C_alpha _and C_1_' atoms;

2., The option to exclude neighbor atoms and to improve the specificity of the method;

3. The direct connection to a Residue Contact Table which automatically provides a basic statistical analyzes of the residue co-locations.

It is expected, that statistical analyses of residue co-locations in protein and nucleic acid sequences will provide further insight and understanding the rules of macromolecular interactions. The ultimate goal of these types of studies is to find short "complementary" or "compatible" sequences/motifs even for specific nucleic acid – protein and protein – protein interactions, something similar to the well known Watson-Crick rules of specific nucleic acid – nucleic acid contacts.

It is well known that in studies of protein interactions, protein engineering and drug design the most important are the interactions between side chains. However, the recent SeqX program is a general purpose tool (for nucleic acids as well as for proteins) for statistical analyzes and visualization of entire-residue co-locations and it does not pay particular attention to side chains and the pattern of the side chain interactions. It does not limit the usefulness of this tool for its original purpose: any significant residue co-locations (i.e. that which are different from random) are necessarily caused by the side chains ('R' in amino acids, 'bases' in nucleic acids) because they are the variable elements of the structures. However a future implementation might focus on analyzes of side chain to side chain co-locations and examine whether that will improve the specificity of this tool.

## Conclusion

The SeqX is a simple, easy to use specialized tool for visualization and statistical analyses of protein and nucleic acid residue co-locations. It is mainly and specifically developed to study known and novel specific residue interactions.

## Authors' contributions

JCB designed and tested the tool, and wrote this article. GF implemented the software. GF is the winner of the first prize of the First Hungarian George Gamow Competition and Fellowship in 2004 with his contribution.

## Supplementary Material

Additional File 1 SeqX_1.041_05601.jar. see this article Click here for file
